# Supplementary material for: Structural and biochemical characterisation of the Providencia stuartii arginine decarboxylase shows distinct polymerisation and regulation
Source: Commun Biol. 2022 Apr 5;5:317. doi: 10.1038/s42003-022-03276-1 (PMC8983666; doi:10.1038/s42003-022-03276-1)
Supplement: Supplementary file 2 — Supplementary Information [file 42003_2022_3276_MOESM2_ESM.pdf]

Supplementary Figure 1.

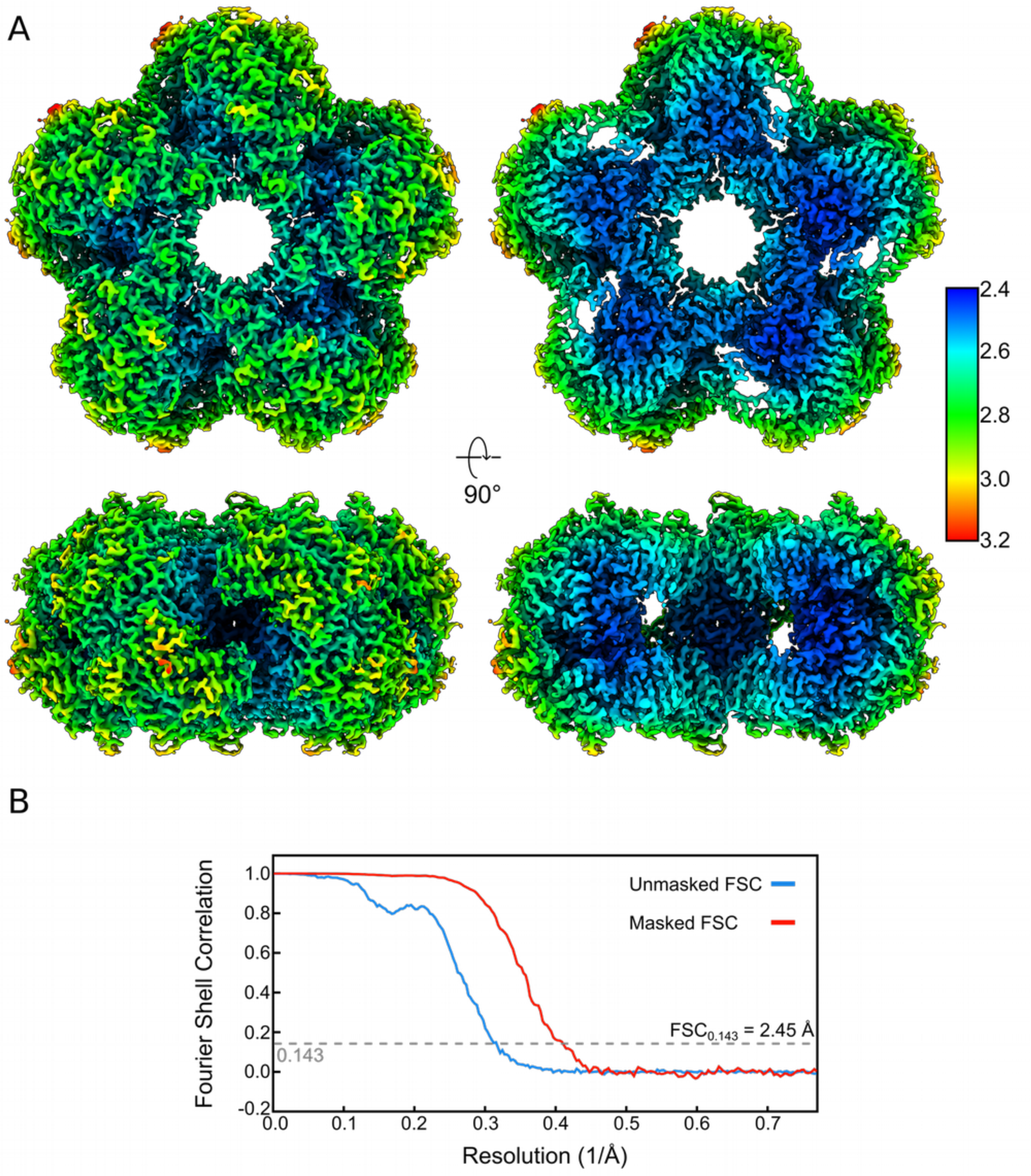

Supplementary Figure 1. Assessment of the resolution of the cryo-EM map of the *P. stuartii* Adc decamer. A) Cryo-EM map of the Adc decamer coloured according to local resolution shown from the top (above) and side (below). The map is shown both in full (left) and clipped (right). **B)** Gold-standard Fourier Shell Correlation (FSC) curves for both unmasked (blue) and masked (red) maps. The 0.143 cut-off is indicated by the dotted line.

Supplementary Figure 2.

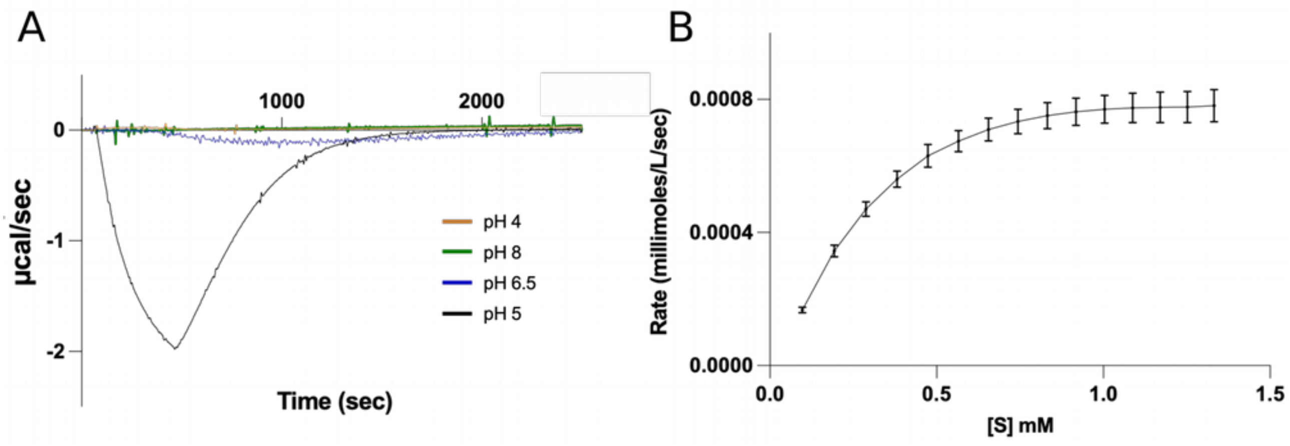

Supplementary Figure 2. Kinetics of *P. stuartii* Adc enzymatic activity determined by Isothermal Titration Calorimetry at different pHs. A) ITC experiments carried out in single injection mode at different pH values (pH 8 = brown, pH 6.5 = green, pH 5 = tan, pH 4.5 = blue) showing that Adc is only active at pH 5. **B)** Arginine decarboxylase activity at pH 5. Error bars correspond to the standard deviation from triplicate experiments. The enzymatic parameter estimates within the 95% confidence interval are K_M=0.331 mM (0.28, 0.38) and k_{cat}=102 sec^{-1} (97, 108).

Supplementary Figure 3.

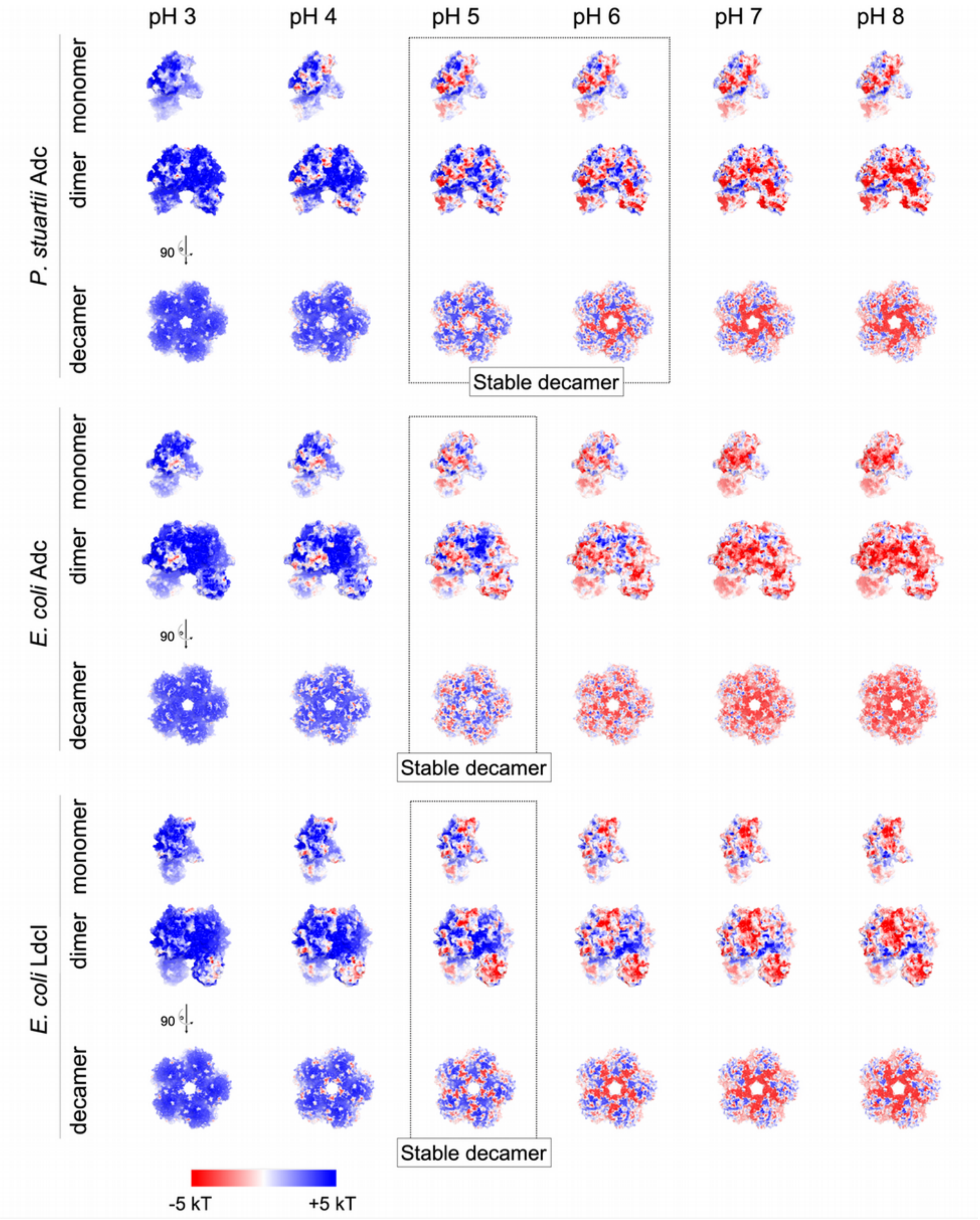

Supplementary Figure 3. Charge distribution at the surface of *P. stuartii* Adc, *E. coli* Adc and *E. coli* LdcI monomers, dimers and decamers calculated at various pHs. For the three oligomers formed by each protein, the electrostatic potential at ± 5 kT is mapped onto the Connolly surface. The pH at which decamers and/or stacks were found to be stable experimentally are indicated. Collapse of these oligomers is likely to stem from electrostatic repulsion between monomers at either extremely acidic or close-to-neutral pH.

Supplementary Figure 4.

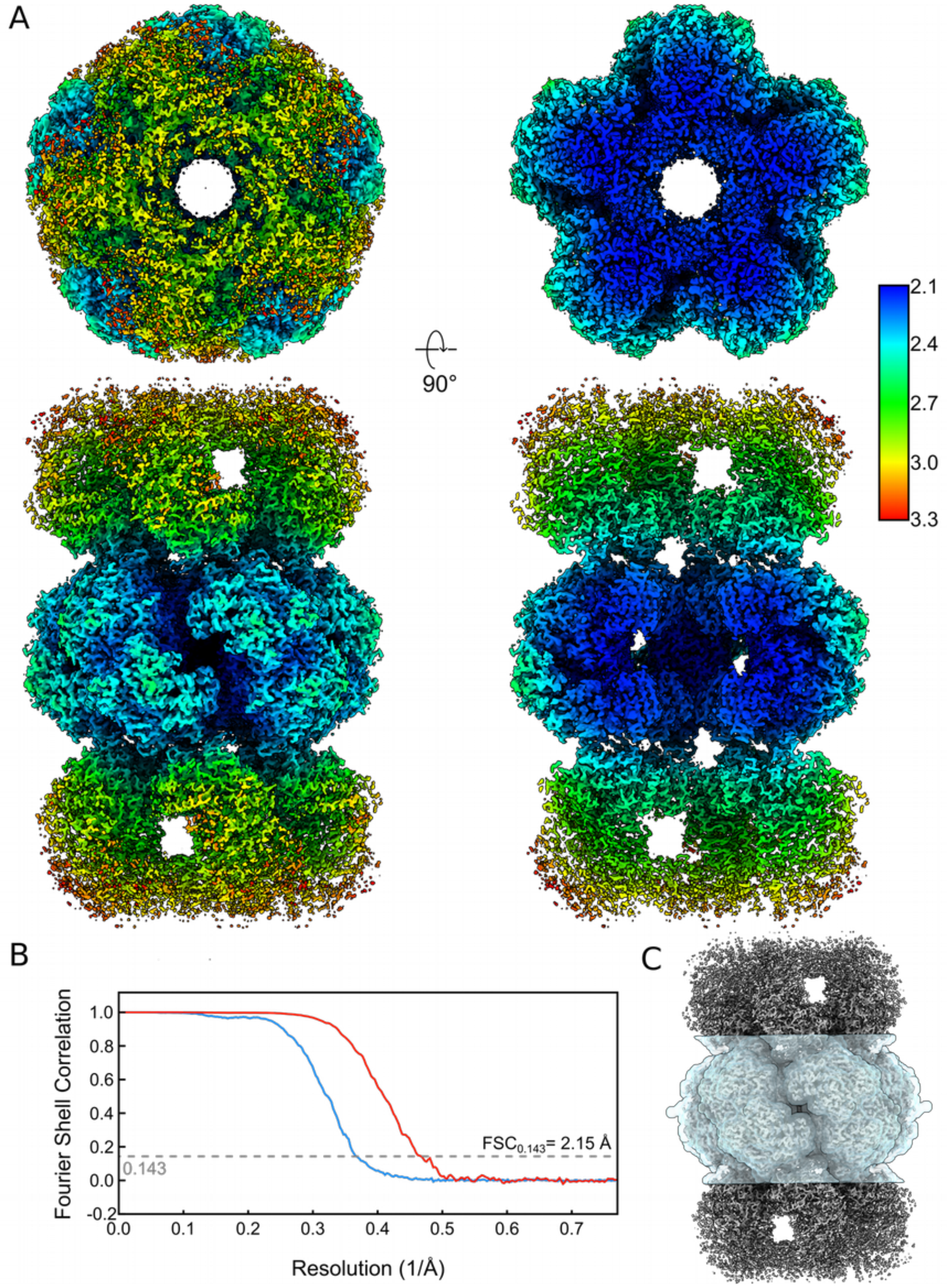

Supplementary Figure 4. Assessment of the resolution of the cryo-EM map of the *P. stuartii* Adc stack. A) Full unmasked cryo-EM map of the Adc stack coloured according to local resolution shown from the top (above) and side (below). The map is shown both in full (left) and clipped (right). **B)** Gold-standard Fourier Shell Correlation (FSC) curves for unmasked (blue) and masked (red) maps. The 0.143 cut-off is indicated by the dotted line. **C)** Mask used for 3D refinement and the calculation of global resolution, shown in transparent blue over the unmasked volume. The mask encloses only the central decamer and the inter-decamer interface regions to negate the relative long-range disorder of the stacks.

Supplementary Figure 5.

A

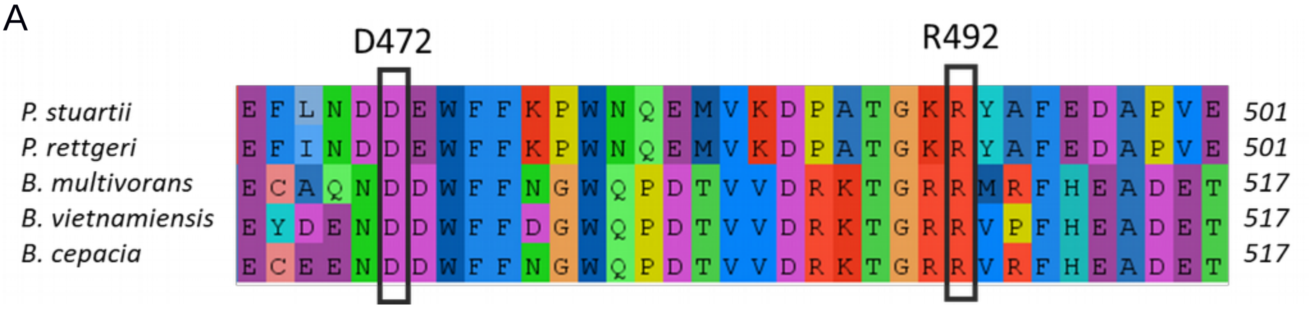

B

Adc	*P. stuartii*	*P. rettgeri*	*B. multivorans*	*B. vietnamiensis*	*B. cepacia*
P. stuartii	100.00	95.63	48.61	48.47	48.21
P. rettgeri	95.63	100.00	49.14	49.01	49.01
B. multivorans	48.61	49.14	100.00	93.20	94.22
B. vietnamiensis	48.47	49.01	93.20	100.00	94.99
B. cepacia	48.21	49.01	94.22	94.99	100.00

Supplementary Figure 5. Conservation of the structural determinants of Adc polymerisation. A) Sequence alignment showing high conservation of the structural determinants of Adc stack formation in *P. stuartii*, *P. rettgeri, Burkholderia cepacia*, *B. multivorans* and *B. vietnamiensis.* The stack-forming residues D472 and R492 of *P. stuartii* (WP_206084812.1) and *P. rettgeri* (WP_125891638.1) are conserved in their respective homologues in *B. cepacia* (WP_048804479.1), *B. multivorans* (WP_014898692.1), *B. vietnamiensis* (WP_014725479.1) and indicated by boxes numbered 1 and 2 respectively. Alignment was performed using AliView. B) Percent Identity Matrix of Adc sequences of *P. stuartii*, *P. rettgeri*, *B. cepacia*, *B. multivorans* and *B. vietnamiensis*. Conservation of stack forming residues D472 and R492 is observed despite the relatively modest identity and phylogenetic distance between these species.

Supplementary Figure 6.

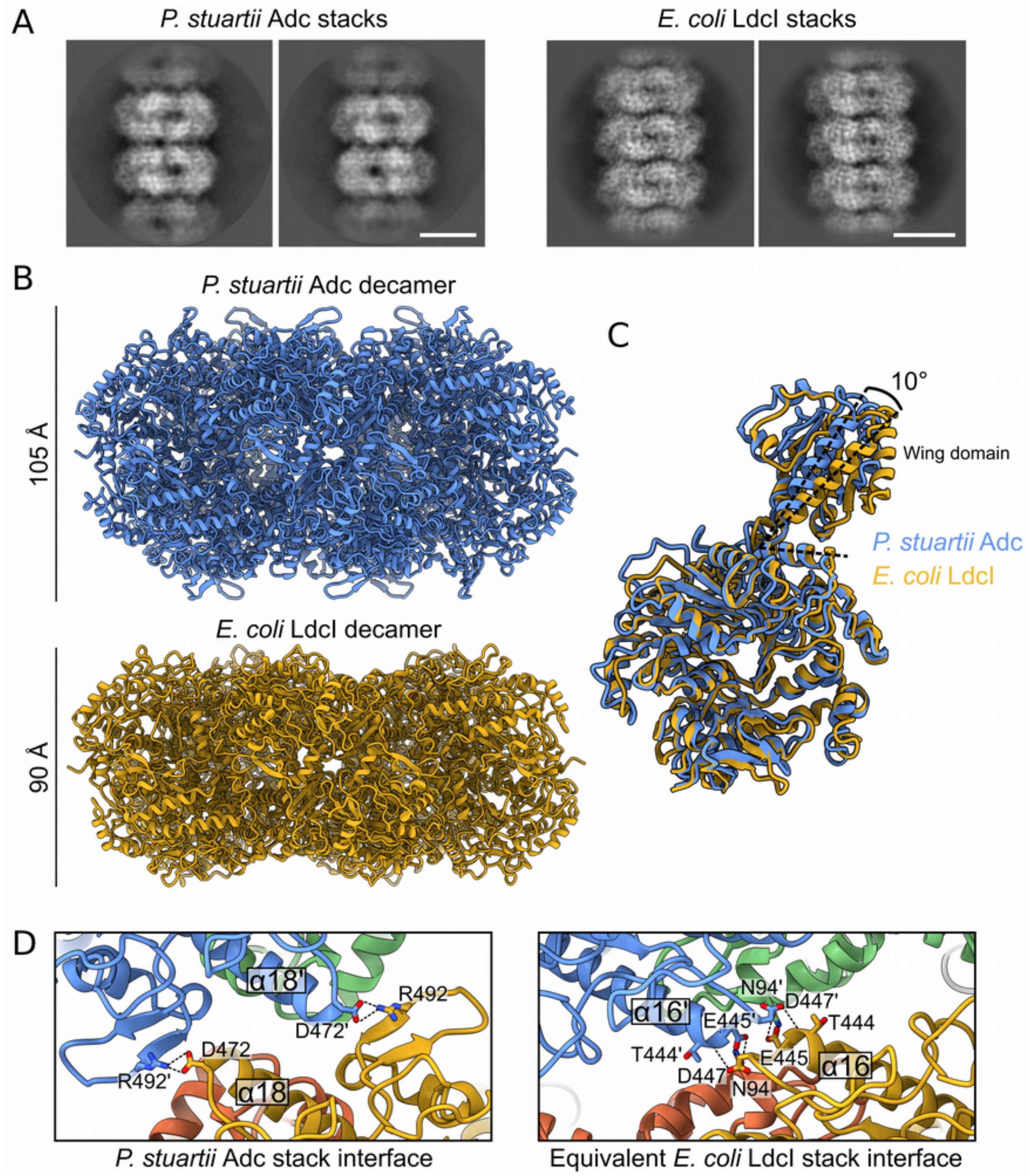

Supplementary Figure 6. Comparison between *P. stuartii* Adc and *E. coli* LdcI structures. A) 2D class averages of *P. stuartii* Adc (left) and *E. coli* LdcI (right) decamer stacks. The rotation between decamers in the Adc stacks is apparent, whereas for LdcI stacks there is negligible rotation between decamers along the stack. There is also a smaller gap between decamers in the LdcI stack compared to the Adc stack. Scale bar = 100 Å. **B)** Decamer structures of *P. stuartii* Adc (above) and *E. coli* LdcI (below), with height measurements on the left. The inserted β-hairpin at the top and bottom of the Adc decamer increases the height of the decameric ring by 15 Å. **C)** Overlaid monomer structures of *P. stuartii* Adc and *E. coli* LdcI, highlighting the 10° difference in the Wing domain angle. **D)** Close-up view of equivalent inter-decamer interfaces in Adc (left) and LdcI (right, termed 'interface 2' as per Figure 3B). The equivalent helices which contribute residues to the interface (α18 in Adc, α16 in LdcI) are labelled, as are stack-forming residues.

Supplementary Figure 7.

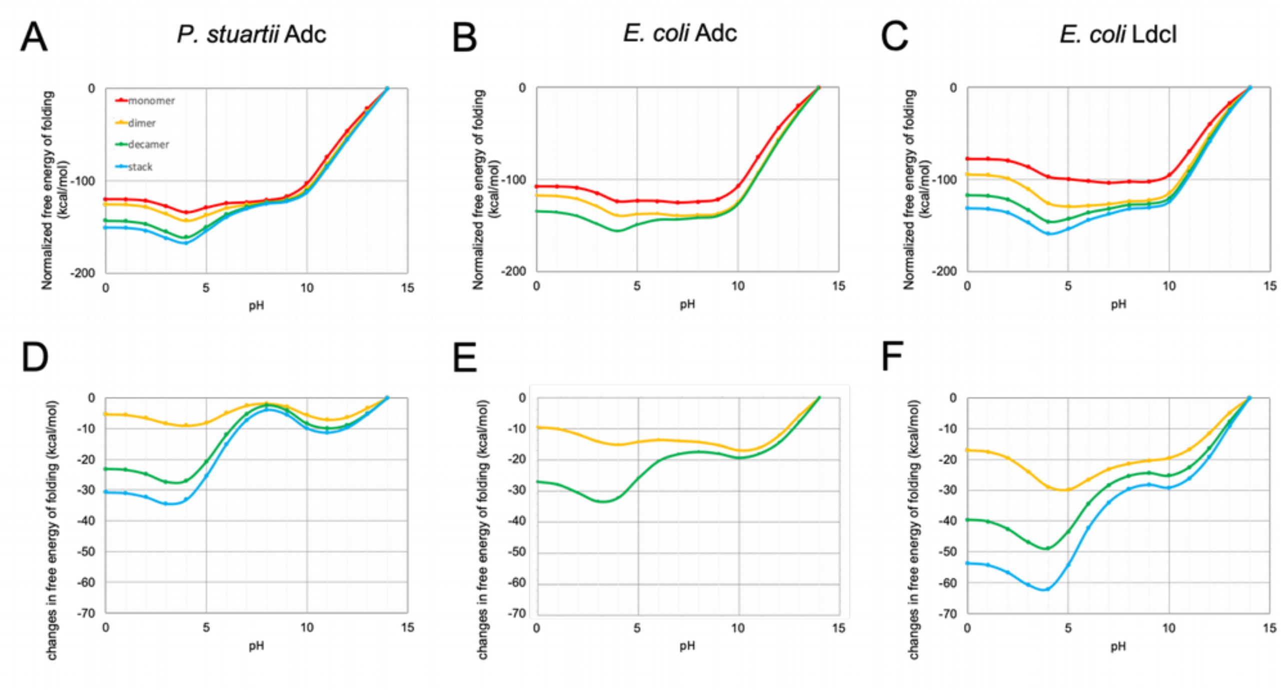

Supplementary Figure 7. pH-dependence of the predicted free energies of folding for the monomer, dimer, decamer and stack formed by *P. stuartii* Adc, *E. coli* Adc and *E. coli* LdcI. A), B), C) Normalized free energy of folding as a function of pH for the various oligomers formed by *P. stuartii* Adc (A), *E. coli* Adc (B) and *E. coli* LdcI (C). **D), E), F)** Corresponding changes in free energy of folding upon transition from the monomer to the dimer, decamer or stack state in *P. stuartii* Adc (D), *E. coli* Adc (E) and *E. coli* LdcI (F). See Supplementary Data 1 for extended analysis.

Supplementary Table 1.

Species	Protein Name	NCBI Ref. Sequence	Nb. of Conserved Residues	N314	D316	E344	G352	R468	D470	E482	Y485	N94	R97	T444	E445	S446	D447
Synthetic *Escherichia coli* C231.deltaA	LdcI	AGX36043.1	14	N	D	E	G	R	D	E	Y	N	R	T	E	S	D
Escherichia albertii	LdcI	WP_025238067.1	14	N	D	E	G	R	D	E	Y	N	R	T	E	S	D
Escherichia coli	LdcI	NP_418555.1	14	N	D	E	G	R	D	E	Y	N	R	T	E	S	D
Escherichia fergusonii	LdcI	WP_002431488.1	14	N	D	E	G	R	D	E	Y	N	R	T	E	S	D
Enterobacter lignolyticus	LdcI	WP_013365210.1	13	N	D	E	G	R	D	E	Y	N	R	G	E	S	D
Klebsiella sp	LdcI	WP_013365210.1	13	N	D	E	G	R	D	E	Y	N	R	G	E	S	D
Klebsiella pneumoniae	LdcI	WP_002892486.1	13	N	D	E	G	R	D	E	Y	N	R	G	E	S	D
Klebsiella variicola	LdcI	WP_012968785.1	13	N	D	E	G	R	D	E	Y	N	R	G	E	S	D
Klebsiella oxytoca	LdcI	WP_004848492.1	13	N	D	E	G	R	D	E	Y	N	R	G	E	S	D
Serratia sp	LdcI	WP_033635725.1	12	N	D	K	G	R	D	E	Y	N	R	V	E	S	D
Serratia marcescens	LdcI	WP_025304020.1	12	N	D	K	G	R	D	E	Y	N	R	V	E	S	D
Enterobacter aerogenes	LdcI	YP_004592843.1	12	N	D	E	G	R	D	E	Y	N	R	G	E	S	E
Salmonella enterica	LdcI	WP_001100652.1	11	N	D	Q	D	R	D	E	Y	N	R	S	E	S	D
Enterobacter lignolyticus	LdcI	WP_013367489.1	11	N	D	E	G	R	D	E	S	N	H	G	E	S	D
Serratia fonticola	LdcI	WP_024485570.1	11	N	D	K	G	R	D	E	Y	N	R	V	E	S	E
Salmonella bongori	LdcI	WP_001100654.1	11	N	D	Q	D	R	D	E	Y	N	R	S	E	S	D
Serratia proteamacula	LdcI	WP_012146472.1	11	N	D	K	G	R	D	E	Y	N	R	V	E	S	E
Yersinia ruckeri	LdcI	WP_038240777.1	11	N	D	K	G	R	D	E	Y	N	R	V	E	S	E
Raoultella ornithinolyt	LdcI	WP_004858936.1	11	N	D	A	G	R	D	E	Y	N	R	G	E	S	E
Yersinia ruckeri	LdcI	WP_038240777.1	11	N	D	K	G	R	D	E	Y	N	R	V	E	S	E
Serratia liquefaciens	LdcI	WP_020828344.1	10	N	D	K	G	K	D	E	Y	N	R	V	E	S	E
Pluribacter gergoviae	LdcI	WP_043082510.1	10	N	D	A	G	R	D	E	Y	N	R	A	E	A	E
Hafnia alvei	LdcI	WP_025800207.1	8	N	D	K	G	D	K	D	Y	N	R	S	E	S	E
Edwardsiella anguillarum	LdcI	WP_034165623.1	7	N	D	Q	E	N	R	D	Y	S	R	T	E	S	E
Hafnia alvei	LdcI	WP_025798687.1	7	N	E	Q	E	N	R	D	Y	S	R	T	E	S	D
Vibrio alginolyticu	LdcI	WP_005481757.1	7	N	Q	E	E	D	S	N	Y	T	R	A	E	S	D
Vibrio parahaemolyt	LdcI	WP_005481757.1	7	N	Q	E	E	D	S	N	Y	T	R	A	E	S	D
Vibrio vulnificus	LdcI	WP_011079948.1	7	N	Q	E	E	D	N	D	Y	T	R	G	E	S	D
Vibrio antiquarius	LdcI	WP_012841314.1	7	N	Q	E	E	D	S	N	Y	T	R	A	E	S	D
Vibrio campbellii	LdcI	WP_010444363.1	7	N	Q	E	E	D	S	N	Y	T	R	A	E	S	D
Aeromonas salmonicida	LdcI	WP_012550963.1	7	N	Q	E	E	D	K	D	Y	T	R	S	E	S	D
Vibrio fischeri	LdcI	YP_205440.1	7	N	Q	E	E	D	K	D	Y	T	R	S	E	S	D
Edwardsiella piscicida	LdcI	WP_012847161.1	7	N	D	Q	E	N	R	D	Y	S	R	T	E	S	E
Edwardsiella sp	LdcI	WP_034165623.1	7	N	D	Q	E	N	R	D	Y	S	R	T	E	S	E
Shimwellia blattae	LdcI	WP_002441073.1	7	N	D	K	D	S	D	N	Y	S	R	G	E	S	E
Edwardsiella ictaluri	LdcI	WP_015872623.1	7	N	E	K	G	D	Q	N	Y	G	R	S	E	S	D
Edwardsiella tarda	LdcI	WP_047059123.1	6	N	E	R	E	E	N	N	Y	G	R	A	E	S	D
Edwardsiella anguillarum	LdcI	WP_034164754.1	6	N	E	Q	E	D	K	N	Y	G	R	S	E	S	D
Edwardsiella piscicida	LdcI	WP_012847625.1	6	N	E	K	G	D	K	D	F	S	R	S	E	S	D
Vibrio cholerae	LdcI	WP_001019828.1	6	N	Q	E	E	D	N	N	Y	T	R	G	E	S	E
Edwardsiella ictaluri	LdcI	WP_015870265.1	6	N	E	K	G	D	K	D	F	S	R	S	E	S	D
Edwardsiella sp	LdcI	WP_034163233.1	6	N	E	K	G	D	K	D	F	S	R	S	E	S	D
Edwardsiella anguillarum	LdcI	WP_034163233.1	6	N	E	K	G	D	K	D	F	S	R	S	E	S	D
Edwardsiella sp	LdcI	WP_034164754.1	6	N	E	Q	E	D	K	N	Y	G	R	S	E	S	D
Edwardsiella piscicida	LdcI	WP_015462316.1	6	N	E	Q	E	D	K	N	Y	G	R	S	E	S	D
Edwardsiella ictaluri	LdcI	WP_015869742.1	6	N	G	Q	E	N	R	D	Y	S	R	T	E	S	E
Pluralibacter gergoviae	LdcI	WP_043082011.1	5	N	N	D	D	N	R	D	F	N	R	S	E	S	E
Shimwellia blattae	LdcI	WP_002439058.1	5	N	D	K	D	N	R	N	Y	S	Q	A	E	S	E
Edwardsiella tarda	LdcI	WP_005280999.1	5	N	D	Q	E	N	R	D	F	S	R	S	E	S	E
Edwardsiella tarda	LdcI	WP_005282982.1	5	N	E	K	D	D	K	N	F	S	R	S	E	S	D
Moritella viscosa	LdcI	WP_045112309.1	5	N	Q	E	E	D	K	N	Y	T	R	A	Q	S	E
Aeromonas hydrophila	LdcI	YP_855733.1	5	N	G	E	E	D	K	D	Y	T	R	E	Q	S	E
Aeromonas veronii	LdcI	WP_005349824.1	5	N	G	E	E	D	K	N	Y	T	R	D	Q	S	E
Aeromonas salmonicida	LdcI	WP_005312101.1	5	N	G	E	D	D	K	D	Y	T	R	D	Q	S	E
Aeromonas schubertii	LdcI	WP_060586925.1	5	N	G	E	D	D	K	D	Y	T	R	E	Q	S	E
Enterobacteriaceae strain FGI57	LdcI	WP_015964930.1	3	N	D	K	T	T	G	G	F	D	G	A	S	S	E

Supplementary Table 1. Conservation of interface residues in Cluster 2 LdcIs

Supplementary Table 2.

Ammonia	*Df*	*Deviance*	*Resid.Df*	*Resid. Dev*	*F*	*Pr(>F)*	*Significance*
NULL			25	1046083			
Condition	1	138843	24	907240	7.923	0.0114676	*
Dose	3	521700	21	385540	9.9235	0.0004385	***
Condition:Dose	3	71507	18	314034	1.3602	0.2866969	
Urea	*Df*	*Deviance*	*Resid.Df*	*Resid. Dev*	*F*	*Pr(>F)*	*Significance*
NULL			25	909583			
Condition	1	467056	24	442527	23.656	0.0001248	***
Dose	3	90033	21	352494	1.52	0.2433957	
Condition:Dose	3	8426	18	344068	0.1423	0.9332977	
pH	*Df*	*Deviance*	*Resid.Df*	*Resid. Dev*	*F*	*Pr(>F)*	*Significance*
NULL			31	561946			
Condition	1	121644	30	440302	11.7639	0.002394	**
Dose	4	142179	26	298122	3.4374	0.025073	*
Condition:Dose	4	72771	22	225351	1.7594	0.173095	

Supplementary Table 2. GLM statistics on *adc* expression data. Significance codes: 0 ‘***’ 0.001 ‘**’ 0.01 ‘*’ 0.05 ‘.’ 0.1 ‘ ’ 1. ‘Df’ indicates the degrees of freedom, ‘Resid.Df’ the residual degrees of freedom, ‘Resid.Dev’ the residual deviation of the model, ‘F’ the value of the Fisher’s test; ‘Pr(>F)’ the p-value of the statistical test. For each treatment (‘Ammonia’, ‘Urea’ and ‘pH’), ‘NULL’ refers to the values of the null model, ‘Condition’ to the effect of the bacteria social structure (FCC or SAB), ‘Dose’ to the effect of the concentration (0, 100, 500 and 1000 mM for ammonia and urea or 5, 6, 7, 8, 9 for pH). ‘Condition:Dose’ refers to the interaction between the two abovementioned parameters.
