# Supplementary material for: Structural and biochemical characterisation of the Providencia stuartii arginine decarboxylase shows distinct polymerisation and regulation
Source: Commun Biol. 2022 Apr 5;5:317. doi: 10.1038/s42003-022-03276-1 (PMC8983666; doi:10.1038/s42003-022-03276-1)
Supplement: Supplementary file 3 — Description of Additional Supplementary Files [file 42003_2022_3276_MOESM3_ESM.pdf]

## Description of Additional Supplementary Files

**File name: Supplementary Data 1**

**Description:** Extensive structural analysis. a) Analysis of dimer, decamer and, when applicable, stack interfaces in *P. stuartii* Adc isolated and stacked decamers*, E. coli* Adc decamers and *E. coli* LdcI stacked decamers. b) Extensive characterisation of charged residues at various packing interfaces for *P. stuartii* Adc*, E. coli* Adc and *E. coli* LdcI. c) Changes in protein charge, free energy of folding and normalised free energy of folding as a function of pH.

**File name: Supplementary Data 2**

**Description:** Conservation of the stack forming residues in *P. stuartii* and *P. rettgeri*

**File name: Supplementary Data 3**

**Description:** Source data for Figure 1d

**File name: Supplementary Data 4**

**Description:** Source data for Figure 4
