# Supplementary material for: Structural and biochemical characterisation of the Providencia stuartii arginine decarboxylase shows distinct polymerisation and regulation
Source: Commun Biol. 2022 Apr 5;5:317. doi: 10.1038/s42003-022-03276-1 (PMC8983666; doi:10.1038/s42003-022-03276-1)
Supplement: Supplementary file 8 — Reporting Summary [file 42003_2022_3276_MOESM8_ESM.pdf]

nature portfolio

Corresponding author(s): Irina Gutsche

Last updated by author(s): Feb 1, 2022

Reporting Summary

Nature Portfolio wishes to improve the reproducibility of the work that we publish. This form provides structure for consistency and transparency in reporting. For further information on Nature Portfolio policies, see our Editorial Policies and the Editorial Policy Checklist.

Statistics

For all statistical analyses, confirm that the following items are present in the figure legend, table legend, main text, or Methods section.

n/a	Confirmed	
☐	☒	The exact sample size (*n*) for each experimental group/condition, given as a discrete number and unit of measurement
☐	☒	A statement on whether measurements were taken from distinct samples or whether the same sample was measured repeatedly
☐	☒	The statistical test(s) used AND whether they are one- or two-sided<br>*Only common tests should be described solely by name; describe more complex techniques in the Methods section.*
☐	☒	A description of all covariates tested
☐	☒	A description of any assumptions or corrections, such as tests of normality and adjustment for multiple comparisons
☐	☒	A full description of the statistical parameters including central tendency (e.g. means) or other basic estimates (e.g. regression coefficient) AND variation (e.g. standard deviation) or associated estimates of uncertainty (e.g. confidence intervals)
☐	☒	For null hypothesis testing, the test statistic (e.g. *F*, *t*, *r*) with confidence intervals, effect sizes, degrees of freedom and *P* value noted<br>*Give P values as exact values whenever suitable.*
☒	☐	For Bayesian analysis, information on the choice of priors and Markov chain Monte Carlo settings
☒	☐	For hierarchical and complex designs, identification of the appropriate level for tests and full reporting of outcomes
☒	☐	Estimates of effect sizes (e.g. Cohen's *d*, Pearson's *r*), indicating how they were calculated

Our web collection on statistics for biologists contains articles on many of the points above.

Software and code

Policy information about availability of computer code

Data collection	EPU
Data analysis	CryoSPARC, RELION, Chimera, PHENIX, Coot, PROPKA, PDB2PQR, APBS, Pymol, Refeyn AcquireMP, Refeyn DiscoverMP, WebLogo Software, BLAST, R

For manuscripts utilizing custom algorithms or software that are central to the research but not yet described in published literature, software must be made available to editors and reviewers. We strongly encourage code deposition in a community repository (e.g. GitHub). See the Nature Portfolio guidelines for submitting code & software for further information.

Data

Policy information about availability of data

All manuscripts must include a data availability statement. This statement should provide the following information, where applicable:

- Accession codes, unique identifiers, or web links for publicly available datasets
- A description of any restrictions on data availability
- For clinical datasets or third party data, please ensure that the statement adheres to our policy

Cryo-EM maps, along with the corresponding fitted atomic structures, have been submitted to the EMDB and PDB with accession codes EMD: 13261 and PDB: 7P9B for P. stuartii Adc decamers and EMD: 13466 and PDB: 7PK6 for Adc stacks.

Field-specific reporting

Please select the one below that is the best fit for your research. If you are not sure, read the appropriate sections before making your selection.

☒ Life sciences ☐ Behavioural & social sciences ☐ Ecological, evolutionary & environmental sciences

For a reference copy of the document with all sections, see nature.com/documents/nr-reporting-summary-flat.pdf

Life sciences study design

All studies must disclose on these points even when the disclosure is negative.

Sample size	For Cryo-EM analysis - 268,579 particles for the Adc stack, 303,475 particles for the Adc decamer
Data exclusions	For Cryo-EM analysis - the best 268,579 particles were retained for 3D refinement for the Adc stack, and the best 46,854 partcles for the Adc decamer were retained.
Replication	Multiple measurements were taken for biophysical analyses and adc expression quantification, multiple refinements were run for cryo-EM analysis.
Randomization	For cryo-EM analysis, refinement was carried out according to the gold-standard refinement protocol, which involves random assignment of particles to half-datasets.
Blinding	Not applicable - not relevant to the methods used.

Reporting for specific materials, systems and methods

We require information from authors about some types of materials, experimental systems and methods used in many studies. Here, indicate whether each material, system or method listed is relevant to your study. If you are not sure if a list item applies to your research, read the appropriate section before selecting a response.

Materials & experimental systems

n/a	Involved in the study	
☒	☐	Antibodies
☒	☐	Eukaryotic cell lines
☒	☐	Palaeontology and archaeology
☒	☐	Animals and other organisms
☒	☐	Human research participants
☒	☐	Clinical data
☒	☐	Dual use research of concern

Methods

n/a	Involved in the study	
☒	☐	ChIP-seq
☒	☐	Flow cytometry
☒	☐	MRI-based neuroimaging
